# Supplementary material for: Content-rich biological network constructed by mining PubMed abstracts
Source: BMC Bioinformatics. 2004 Oct 8;5:147. doi: 10.1186/1471-2105-5-147 (PMC528731; doi:10.1186/1471-2105-5-147)
Supplement: Additional File 2 — The original results of the above study (non-essential files are deleted to keep the file size under the limit set by BMC bioinformatics). [file 1471-2105-5-147-S2.bz2 › chilibotAdditionalFile2/dip20/256ID8242751E1019/html/CDKN1A_CCNA2.html]

 


 **CDKN1A** and **CCNA2** 
  
Found 163 abstracts in PubMed, retrieved 20.  
 

 What does Google say? 
 PDF only 
| .edu only 

---

**Interactive relationship** (e.g. stimulation, inhibition, etc)

**Inhibitory relationship**- p21 Waf1  [ **CDKN1A** ]  Cip1  [ **CDKN1A** ]  exerts a key role in driving this G 2 exit both by inhibiting cyclin B1 Cdk1 and cyclin A  [ **CCNA2** ]  Cdk1 2 complexes, which control G 2 M progression, and by blocking the phosphorylation of pRb family proteins.  Ref: 12881433 EMBO JEMBO J,
**Stimulatory relationship**- Western blot analysis also shows that DHA greatly reduces the level of cyclin A  [ **CCNA2** ] , while increasing the level of p21 WAF1  [ **CDKN1A** ] , a cellular inhibitor of cyclin A  [ **CCNA2** ]  cyclin dependent kinase 2 activity.  Ref: 12492401 Biochem J< MedlineTA>Biochem J,
- Thus, PD selectively elevates p21 WAF1  [ **CDKN1A** ]  CIP1  [ **CDKN1A** ]  levels and thereby arrests the cell cycle at G1 S phase by down regulating Cyclin A  [ **CCNA2** ]  Cdk2 activity.  Ref: 12819186 Carcinogenesis< MedlineTA>Carcinogenesis,
**Neutral relationship**- Highly potent p21 WAF1  [ **CDKN1A** ]  derived peptide inhibitors of CDK mediated pRb phosphorylation delineation and structural insight into their interactions with cyclin A  [ **CCNA2** ] .  Ref: 12383116 J Pept Res, 2002

**Non-interactive relationship** (e.g. studied together, co-existance, homology, etc.)

- Similarly, levels of the p21 WAF1  [ **CDKN1A** ]  CIP1  [ **CDKN1A** ]  protein that is associated with the Cyclin A  [ **CCNA2** ]  Cdk2 complex increased, and these increases correlated well with the down regulation of Cyclin A  [ **CCNA2** ]  Cdk2 activity.  Ref: 12819186 Carcinogenesis< MedlineTA>Carcinogenesis, 2002
- Panaxadiol selectively inhibits Cyclin A  [ **CCNA2** ]  associated Cdk2 activity by elevating p21WAF1 CIP1  [ **CDKN1A** ]  protein levels in mammalian cells.  Ref: 12819186 Carcinogenesis< MedlineTA>Carcinogenesis, 2002
- METHODS For each patient, flow cytometry DNA analyses on frozen samples and on immunohistochemical staining were performed, including Ki 67, cyclin A  [ **CCNA2** ] , p53, and p21 waf1  [ **CDKN1A** ]  p21, with assessment of the percentages of positive nuclei were assessed.  Ref: 12627500 Cancer, 2003
- PS 341 exposure in H460 cells led to stabilization of p53, induction of p21 cip  [ **CDKN1A** ]  waf 1  [ **CDKN1A** ]  and MDM2 expression, an increase in cyclin B and cyclin A  [ **CCNA2** ] , and the activation of cyclin B and cyclin A  [ **CCNA2** ]  kinases.  Ref: 12631620 Clin Cancer Res, 2003
- Raf activation increased the expression of cyclin A  [ **CCNA2** ] , cyclin D, cyclin E, and p21 Cip1  [ **CDKN1A** ] , which are associated with G 1 progression.  Ref: 12429936 Cell Cycle< MedlineTA>Cell Cycle, 2003
- The array data enabled us to focus on the role of potential key genes e.g., GADD153, CHK2, p21 WAF1  [ **CDKN1A** ] , cyclin A  [ **CCNA2** ] , CDK1, and DHFR that might be targets of MSA in impeding cell cycle progression.  Ref: 12517777 Cancer Res, 2003
- When cells were synchronized in S phase with aphidicolin and then released, activation of Delta MEKK3 ER\* resulted in the up regulation of p21 CIP1  [ **CDKN1A** ]  and a pronounced inhibition of cyclin A  [ **CCNA2** ]  CDK2 and cyclin B1 CDK1 kinase activity.  Ref: 12444545 Oncogene, 2002
